# Mandela Yoga: a community case study for a post-incarceration reentry service for men of color in recovery

**DOI:** 10.3389/fpubh.2025.1514946

**Published:** 2025-05-09

**Authors:** Richa Gawande, Felipe Kalatauma Rosario, Carlos Santiago, Jeffrey Thomas, Julia Naganuma-Carreras, Tori Blot, Keyona Aviles, Paula Gardiner, Zev Schuman-Olivier

**Affiliations:** ^1^Department of Psychiatry, Harvard Medical School, Boston, MA, United States; ^2^Cambridge Health Alliance, Center for Mindfulness and Compassion, Cambridge, MA, United States; ^3^Mandela Yoga Project, Cambridge, MA, United States

**Keywords:** mindfulness, yoga, post-incarceration health and well-being, substance use recovery, people of color, community-based participatory research

## Abstract

**Background:**

Disparities in substance use treatment access and outcomes between communities with racially, economically, linguistically, and mentally/physically marginalized identities and more privileged populations are staggering. Communities of color lack access to culturally resonant treatment options that incorporate the role of racial oppression, address the chronic effects of stress on the nervous system, provide culturally-and linguistically-matched community support in substance use recovery, and contend with social determinants of health. Mandela Yoga, a community-based peer-led mindfulness intervention, was created to address disparities in health and substance use treatment access among communities of color. Mandela Yoga was co-developed by Black and Brown yoga teachers, therapists, and community leaders with lived experienced of recovery, incarceration, chronic illness, and racism. A Mandela Yoga community reentry services implementation was funded by a Massachusetts Department of Public Health Bureau of Substance Abuse Services grant for overdose risk reduction for people recently released from incarceration.

**Objectives:**

In this community case study, we present a qualitative analysis of a 12-week Mandela Yoga implementation as part of a Federally Qualified Health Center reentry program focused on post-incarceration opioid overdose risk reduction among men of color. Through a community-based participatory approach, we feature the voices and lived experiences of the peer facilitator and a reentry services participant, who are co-authors and shaped the qualitative analysis.

**Methods:**

We documented attendance and conducted interviews with the Mandela Yoga peer facilitator and one participant. Together we conducted a thematic analysis of the interviews to explore key elements that most impacted recovery and healing.

**Results:**

We report on the delivery and attendance of the implementation. We present excerpts illustrating four key themes that emerged from the interviews: (1) Breath and Mind–Body Connection Leads to Presence; (2) Consistency; (3) Peer Connection; (4) Agency and Positive Action.

**Discussion:**

We explore how Mandela Yoga may build recovery capital and the mechanisms by which it may support healing from addiction and trauma in communities of color. We discuss study limitations and considerations for future implementations.

**Conclusion:**

Mandela Yoga shows promise as a mind–body-community intervention for communities of color in recovery and post-incarceration.

## 1 Introduction

*“I see my life with Mandela Yoga…I get to breathe more. I get to sit back and I understand. And I know now that I always have an option.” (PF, Mandela Yoga Peer Facilitator, Federally-Qualified Health Center reentry service)*

The American public health crisis of racism has contributed to substance use disorder (SUD), mental health conditions, incarceration, suicide, overdose, reduced access to and use of community-based SUD treatment for people of color (POC), especially those with multiple oppressed identities (1–7). Despite similar rates of substance use among White individuals, Black, Latino and Indigenous individuals are two to three times as likely to be arrested for illicit drug use (8, 9). Overdose mortality rate is twice as high for Black individuals in counties where income inequality is high, and overdose mortality rates are rising 1.5 times faster for Hispanics than non-Hispanics (10). Black and Hispanic formerly incarcerated individuals often face numerous barriers following incarceration that challenge their abilities to reintegrate into society, especially in the first year of reentry (11).

Race-based stress and the psychological, physical and emotional impacts of intergenerational oppression may play a large role in the initiation and maintenance of SUD among POC (4, 12–14). Racial trauma and oppression play a key role in this public health polycrisis with both biological and socioecological impacts. Lab research demonstrates that social subordination increases substance use self-administration behaviors and leads to neuroplasticity in dopamine receptor function that increases susceptibility to addiction (15). Racist policies impact access to housing, employment, healthcare and policing, medical mistrust, substance use stigma, incarceration rates, disparities in mental and physical health, together acting as barriers for SUD treatment (i.e., geographical barriers, financial constraints). Communities have limited availability of culturally and linguistically effective SUD treatment providers (6). The cumulative impact of these barriers contributes to chronically high allostatic load for POC (16).

During the coronavirus pandemic, rates of fatal drug overdoses increased among Black males who also have higher rates of recidivism upon their release from incarceration (17–19). The Bureau of Substance Addiction Services (BSAS) in Massachusetts sought vendors to provide culturally responsive, wrap-around reentry services to Black and Latino men who were at high risk for opioid overdose while in the process of reintegrating into society after incarceration. Studies have shown that incarcerated individuals who participate in reentry programs that address substance use have better substance use and recidivism outcomes (20). Group-based settings are known to be supportive for those in recovery and may play a particularly effective role for both healing trauma and supporting recovery for POC. Particularly well-adapted to a group model, mind–body interventions may serve as a promising part of substance use treatment. Many traditions across the world have utilized mind–body practices for individual and community-based resilience, social connectedness, and recovery from stress (21–23). The theory of Mindful Self-Regulation proposes that training in mindful awareness can support attention regulation, emotion regulation, and changes in self-awareness, such as interoception and self-compassion (24, 25). Expanding this further, Schmalzl et al. conceptualize that movement-based embodied contemplative practices support a rich, bidirectional signaling between the body and the brain across several networks involved in allostatic load, vagal tone, and self-conceptualization through the default mode network (26). These elements may be particularly helpful for trauma recovery and the cognitive and emotional flexibility and motivation required in restructuring of the sense of self after incarceration. Evidence supporting the impact of mind–body interventions on reducing experiential avoidance, craving, depressive mood, and anxiety in the context of SUD relapse prevention is accumulating (27–29). Members of our team developed a pilot mindful recovery program, combining a mindfulness-based intervention (MBI) with group-based opioid treatment with buprenorphine. This program supported treatment retention with high levels of medication adherence with buprenorphine, while decreasing experiential avoidance and increasing self-compassion (30). When compared to evidence-based group treatment with buprenorphine, the MBI group resulted in greater reduction in opioid craving (31). Group yoga has also shown promise in the context of SUD as a standalone treatment, or as an adjunctive treatment in combination with medication for opioid use disorder, for outcomes of pain and anxiety (32).

Our team and others have found an underrepresentation of racial and ethnic minorities, particularly Black and Indigenous individuals, in mind–body research and practice (33, 34). Research on adapting mind–body interventions for minoritized populations has highlighted barriers such as location, childcare, working multiple jobs, cost, cultural barriers, non-concordance between facilitators and participants in race, ethnicity, language, and lived experiences, stigmas/fears associated with religiosity and spirituality, and the lack of attunement to group-based trauma (35–39). While strengthening the connection between mind and body, few mind–body interventions include the connection with the community and specifically address the impacts of racism and oppression in the larger social world. Interventions designed by communities of color incorporate culturally and linguistically appropriate services and highlight the lived realities of broader historical, social and political contributors to disparities in access and quality of care (21, 22, 34, 36, 40, 41). Combining culturally-resonant group-based mind–body practice with treatment for SUD represents a powerful opportunity for POC. Mandela Yoga Project (MYP) is a Boston-area community non-profit named after Nelson Mandela, who was a South African anti-apartheid activist, politician, and statesman who served 27 years in prison and then served as the first president of South Africa from 1994 to 1999. He won the Nobel Peace Prize and was the country’s first Black head of state with a focus on dismantling the legacy of apartheid by fostering racial reconciliation. Jeffrey Thomas founded MYP after learning how the disproportionate incidence of chronic conditions in his own African American family—of his father’s eight children, three were diabetic, two had a bipolar diagnosis, one was asthmatic—were reflected in society at large. Mr. Thomas wanted to create a program that centers the lived experience of POC and addresses the deleterious and disproportionate outcomes of racism on physical and mental health. The core sequence of the Mandela Yoga intervention is called Sonya’s Sequence, which is named after Mr. Thomas’s sister Sonya, who died in her early 50s from renal failure in the context of uncontrolled Type 2 Diabetes. In contrast, Jeffrey Thomas lives with controlled diabetes as a result of learning yoga during a diabetes group medical visit and then practicing in the community at sites where his sister would not have felt comfortable to attend. His experience of yoga in the group medical visit setting paired with the recognition of a need for other POC to benefit from similar care inspired the co-creation of Mandela Yoga– a gentle, mindful yoga that resourced POC suffering from trauma and illness to be architects in their own healing. Mandela Yoga was co-developed by Black and Brown yoga teachers, therapists, community leaders in recovery and experienced with incarceration, chronic illness and racism. Differentiated from white-culture-centered group contemplative practices, Mandela Yoga is peer-led, which allows participants of color to receive the benefits of group practice augmented by the increased healing benefits of racial, ethnic, and cultural concordance.

In this manuscript, the MY curriculum will be discussed. We then present a community case study featuring two current Hispanic peer facilitators who speak to their direct experiences of the peer-led Mandela Yoga intervention at a reentry services program. The case study explores the Mandela Yoga intervention as a pathway for healing and addiction recovery, and as a means to share leadership and community-building in a Spanish-speaking, Hispanic community. This case study is part of a larger community-based participatory approach to shape the Mandela Yoga intervention, and to understand the impact on peer facilitators and community members who have experienced incarceration and addiction.

## 2 Methods

### 2.1 The Mandela Yoga intervention

#### 2.1.1 Co-creation process

The Mandela Yoga intervention was co-curated by Mr. Thomas (JT), who is African American, with a white trauma-sensitive yoga expert teacher, and an African American licensed mental health counselor (KA) who works in communities of Black and Brown individuals in Boston. During development, the team was informed by the expertise of teachers of yoga and mind–body practices, health care providers, and social sector leaders. The intervention was iterated among Federally Qualified Health Center (FQHC) patients and staff, and participants of the reentry program described here, resulting in 15 + peer facilitators of color with lived experience with addiction recovery, diabetes, high blood pressure, racism, chronic pain, aging, incarceration, and/or experience of being unhoused including the peer facilitator and participant whose voices are featured here.

#### 2.1.2 Major threads

The intervention weaves together several wisdom and knowledge traditions and can be summarized in eight threads (Table 1). Threads 1–4 focus on external supportive and liberating structures (trauma-informed, peer connection, undo internalized oppression, cultural humility/access), and threads 5–8 focus on internal supportive and liberating structures (stress reduction in the body and mind, interoceptive awareness, building strength/balance/flexibility, and compassion/kindness). Informed by the socio-ecological model, the intervention draws on participants’ and peer facilitators’ understandings and reciprocal interactions with their community, culture, illnesses, trauma and addiction recovery (42).

**Table 1:** Eight active threads in the Mandela Yoga intervention.

External/Internal	Thread	Description	Examples
External	1. Trauma-informed	Allows for trust, openness, and sharing. Has strong elements of the 6 SAMHSA Trauma-Informed Principles (88).	Choice of sitting, standing, or visualizing at every pose; Participants’ experience is treated as the teacher; Establishing a relationship with the physical room for safety.
	2. Peer Connection	Normalizes challenge and strengthens social connection and a sense of belonging, which is a key healing factor for communities of color who have experienced oppression.	In-person group; Peer is small “t” teacher; Check-in at end elicits peer wisdom.
	3. Undo Internalized Racism	Addresses the chronic, unconscious impact of internalized racism (89) and oppression (“there’s something wrong with being a person of color” “I’m not deserving of self-worth, good outcomes, health, or safety.”)	“Your presence is healing;” Participants are welcomed in their full humanity by the peer facilitator.
	4. Cultural Humility/Culturally Resonant Practices	Cultural wisdom practice co-designed by the peer facilitator and participants encourages connection to ancestral and cultural homes and may promote growth, curiosity and confidence about one’s own sources of well-being and recovery.	Peer facilitators often teach in their first language in community settings, which participants report is essential for nervous system rest; Culturally responsive prayer, song, food, dialog, gesture is offered.
Internal	5. Stress Reduction	Slow breathing, postures, and the above elements allows patients to learn what rest feels like in the body and has led to self-reported reductions in stress levels and blood pressure. Contrasts the experience of chronic stress and vigilance present for many communities of color, this is a new experience that can be drawn upon anytime once learned.	Check-in with body several times throughout, conscious, purposeful, deep breathing, humming, tapping offers soothing for the vagus nerve/parasympathetic engagement (90)
	6. Interoceptive Awareness	Tuning into body sensations, emotions, and urges in a supportive environment allows for increased self-regulation through exploration and agency about the body and behaviors.	Embodied consent practice invites interoceptive curiosity about how it feels to be offered something you’d really like, something you really do not want, and something you are unsure about; Mindful check-ins
	7. Strength, Balance, and Flexibility	Can build physical, emotional, and physiologic healing.	Psoas engagement, twists, star pose, sun breath, warrior, grounding and standing
	8. Compassion and Kindness	Create opportunities for generosity, kindness and compassion for self and other.	Create opportunities for mutual care, cultural resilience practice, mindful check-ins, group check-ins, and embodied consent practice

**Figure 1:**
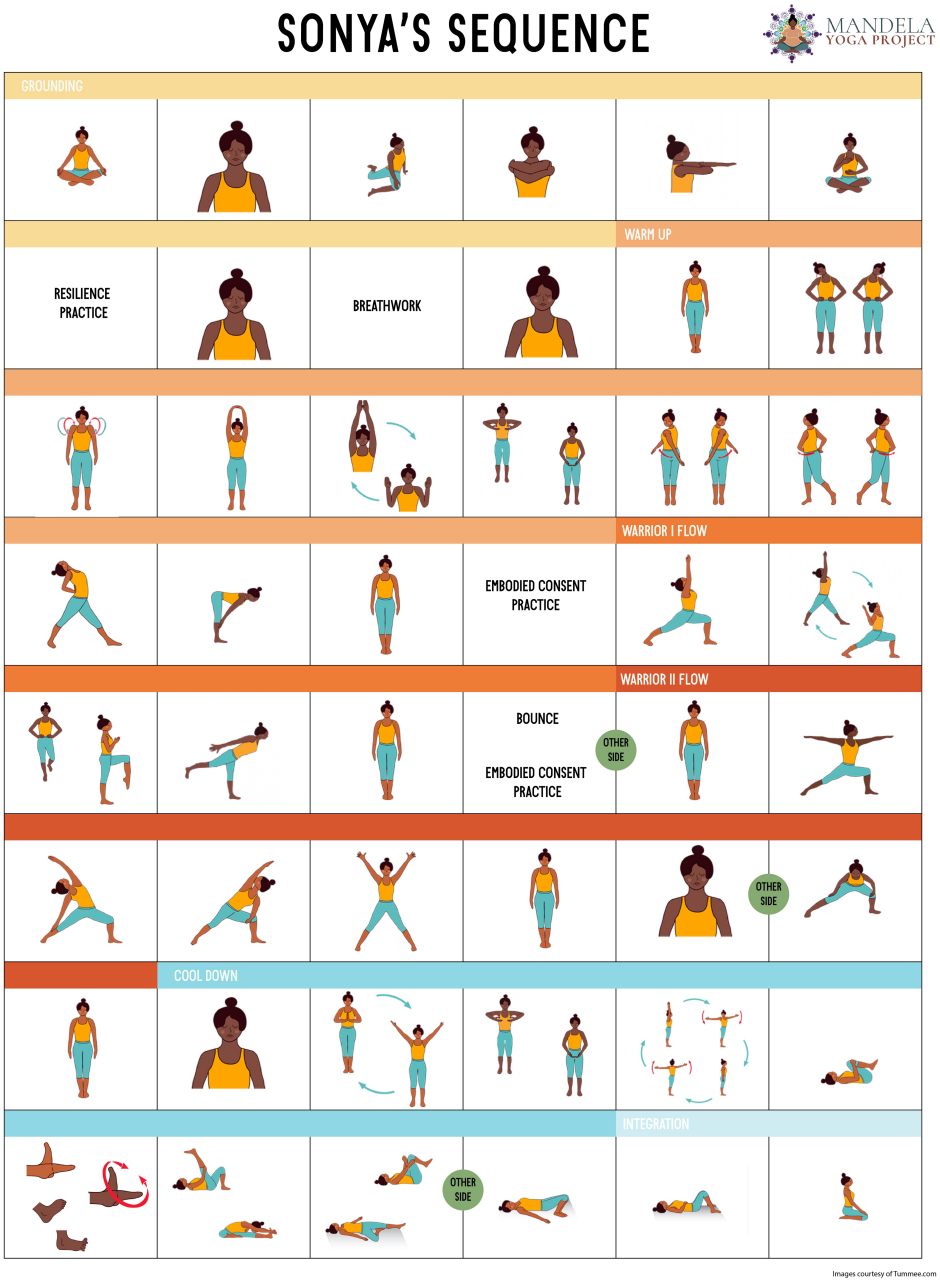
Sonya’s Sequence.

Elements of threads 1–4 include a cultural resilience practice (e.g., song, prayer, dance, gesture, ritual) at the opening and closing of each session, chosen collaboratively by the peer facilitator and the group. A key differentiating element of the Mandela Yoga intervention is that it assumes each participant brings healing assets that is unique to their cultural heritage that can be shared in community. This assumption of cultural assets is not typically emphasized by other contemplative group practices (36). These threads are inspired by best practices from the Trauma-Informed Mind–Body Program curriculum (43), other trauma-informed yoga protocols and anti-oppression protocols (44–48). These include ways to practice agency: learning what “yes” “no,” and “maybe” feel like in the body during the embodied consent practice or considering the invitation to stand, sit, or visualize for each pose. Also included are resilience-building through prolonging positive states (slow purposeful breathing, opportunities for generosity, and kindness to self or other). In threads 5–8, poses were chosen from yoga research protocols for chronic conditions associated with racial disparities such as hypertension, diabetes, chronic pain, and obesity (49–52). A sample class outline can be seen in Table 2. The postural sequence is illustrated in the Mandela Yoga poster (Figure 1), which features bodies of color and underscores the message to POC that “this yoga is for you.” The poster is printed out and shared with participants in each class.

**Table 2:** Sample Mandela Yoga intervention curriculum.

Time	Content	Notes
30–45 min before class	Peer facilitator is present to welcome and check-in with participants informally	Participants are organized in a circle of chairs
0–5 min	Welcome and arriving	Presence and visual seeing
5–10 min	Mindful settling, mindful check-in, look around the space, and humming	Look around the space occurs with psoas engagement, butterfly supportive self-touch and humming (for nervous system support)
10–12 min	Cultural resilience practice	Music, poetry, gesture, dialog, prayer
12–15 min	Hands on belly breathing, standing and grounding	Breath is cued
15–20 min	Neck, shoulder, arm engagement; sun breaths and windmills to take up space	Visualization is always cued as an option
20–25 min	Spinal engagement (static and thoracic twists, cat cow spine)	
25–33 min	Strength and warrior flows; embodied consent practice (x 2)	Yes, No and Maybe are explored and expressed through the mind, body and voice
33–38 min	Cool down (windmills, foot, ankle leg engagement)	
38–43 min	Integrating mindful body scan	
43–45 min	Closing cultural resilience practice	Group go-around, facilitator stays after for individual check-ins

Mandela Yoga is derived from a hatha style of yoga (53), and brings together several elements of evidence-based mind–body interventions, including movement-based embodied contemplative practices as conceptualized by Schmalzl and colleagues (26). A summary of the elements shared between Mandela Yoga and other interventions is shown in Supplementary Table 1. The full intervention includes 13 of the 14 Essential Properties of Yoga (54). It also integrates core elements of Mindfulness-Based Interventions (MBIs) such as the body scan, mindful check-ins that facilitate interoceptive awareness, emotion and attention regulation (24, 28). The intervention aligns with core elements of Mindfulness-Based Programs (MBP) as defined by Crane et al. (e.g., underpinned by a model of human experience which addresses causes of distress and pathways to relieving it, developing a new relationship with experience characterized by present moment focus and an approach orientation, supports the development of greater attentional, emotional and behavioral self-regulation) (55). Mandela Yoga not only brings together several approaches, but adds the major elements of (1) peer facilitation; (2) cultural resilience practices that are collaborated on by the participants, unique to each group; (3) trauma-informed care; and (4) acknowledging and healing from internalized racism (Table 1).

#### 2.1.3 Intervention delivery

The intervention is led by POC peer facilitators with lived experience of racism and at least one other intersecting chronic mental, physical, or structural challenge. Peer facilitators meet regularly with MYP teacher trainer mentors for support and community-building. Peer facilitators in training are paid by the MYP nonprofit to attend the 30-h teacher training. Then, trained teachers are compensated by the MYP nonprofit at $100 + per hour which helps pay for childcare and transportation to the classes. As such, the peer facilitators’ social determinants of health and recovery process are also supported. The intervention has most commonly been offered in a 12-week time-frame based on community feedback about what is required to develop trust and consistency with learning the skills within a community setting, but has also been offered in as few as six sessions or as many as 24 sessions, depending on the setting.

### 2.2 Community implementation case and participants

The Bureau of Substance Addiction Services (BSAS) in Massachusetts was seeking vendors to provide programs for Black and Latino men to engage with resources and activities post-release. An FQHC in a primarily Spanish-speaking immigrant metro-Boston community received a 3-year award and contract to offer intensive case management (assessment and individual service planning), linkages to care, care coordination, and reintegration activities. A 12-week Mandela Yoga intervention was offered as part of the reintegration activities. Feasibility outcome for the reentry training was defined as % attending at least 8/12 (66%) sessions. Participants who attended at least 8/12 sessions were offered a spot in the paid Mandela Yoga teacher training program, as per the Mandela Yoga peer facilitator employment model. The feasibility of the peer teacher training model was defined as >66% of those completers who were offered a spot in the MY teacher training program following through and becoming certified to be a peer facilitator. This community case study features interviews with PF and one of the reentry services participants who are both currently engaged with the MYP nonprofit and are co-authors of this manuscript. The peer facilitator (PF) and the former participant who is now a peer facilitator apprentice (referred to here as PFA) shaped the interview content, thematic analysis interpretation, and the focus of this manuscript. Verbal consent was obtained for audio-and video-recording collaborators PF and PFA for the interviews, transcription, analysis, and publishing excerpts. PF and PFA contributed to the manuscript development, and reviewed and approved the final content shared. Ethical review and approval was not required for the study on human subjects since PF and PFA were collaborators and co-authors on the manuscript and were not research participants.

### 2.3 Community implementation interview methods

The interviews were organized around a community participatory and quality improvement approach rather than around the presentation of a clinical case. For the interviews, authors RG and JNC had an introductory conversation with PF and PFA, and then RG interviewed PF and PFA independently. The following topical anchors were used, which were grouped into three categories – autobiographical, Mandela Yoga for Self and Community based primarily on Mandela Yoga Threads 1–4 (see Table 1), and the theoretical framework for acceptability (56) as applied to recovery. These were initial anchors chosen before the interviews, and were secondary to the topics PF and PFA were moved to talk about, which were the primary anchor for the conversations.

Autobiographical

1. When and why did you come to Mandela Yoga?

Mandela Yoga for Self and Community

1. What did it feel like to be in the Mandela Yoga space, was it/how was it different from other spaces
2. What are the most important things that the Mandela Yoga intervention offers to your community?
3. What are you most proud of in your work with Mandela Yoga?

Theoretical Framework for Acceptability as Applied to Recovery

1. Did anything in Mandela Yoga help with/influence your recovery?
2. What are the most important things that Mandela Yoga offers to you?
3. What about the program caused you and your participants to experience change? What lessons have you learned?
4. Were there any challenges you overcame to come to classes/what allowed you to keep coming back?

The interview with PF was in English, on Zoom, lasting 60 min, and audio-and video-recorded through Zoom. The interview with PFA was in-person at the FQHC, lasting about 90 min, and audio-recorded by phone. PFA speaks both Spanish and English; MYP hired for this interview an independent interpretation service that had previously offered interpretation for the MYP teacher training. The professional interpreter also works full-time as a medical interpreter and shares Borinquen (now known as Puerto Rican) ancestry and other identities with PFA. The cultural rapport and previous familiarity was an important part of the psychological and cultural safety of the Mandela Yoga ethos. As such, at times the conversation with PFA was free-flowing; the interpreter offered with accuracy the essence of the meaning of what PFA communicated rather than exact precision of translation.

### 2.4 Interview analysis

Interview transcripts were created automatically by Zoom for the interview with PF and double-audited (authors JNC, TB), or transcribed manually and then audited (JNC, TB), with a second auditor (RG) for any areas that did not have clarity. During the aspects of the conversations that related directly to Mandela Yoga, a qualitative analysis was undertaken using Braun and Clarke’s method for reflexive thematic analysis (57). An initial list of codes was produced (RG, JNC, TB), using an inductive (linked to the data themselves rather than prior theory or ideas) approach. Codes were reviewed for key themes which were discussed in a group (RG, TB, JNC, JT). The remainder of the conversation (to do with the time in prison, ancestry, etc.) was then re-visited and any themes that surfaced in both interviews were searched for in these sections. Themes unique to the interview with PF (e.g., the teacher training) or with PFA (e.g., his relationship to God) were retained and were included in the manuscript. RG, JNC, TB, PFA, and PF met together to discuss the key themes and the interpretation, to which PF and PFA added nuance and refinement, and the group collectively decided on how to convey these themes in the manuscript, promoting community-academic partnerships, the incorporation of community ideas, and power-sharing and restoring imbalance in power in the traditional research process (58, 59). Excerpts are presented to highlight PF and PFA’s experience as a community case series, not as a complete qualitative analysis, as the sample size is not big enough to reach saturation (60). Often there were several excerpts from each interview that captured each theme; the most direct and potent one was chosen and presented for brevity.

## 3 Results

### 3.1 Implementation details

The Mandela Yoga intervention was offered biweekly at the FQHC re-entry services to men who were recently released from incarceration. The sessions were offered between 2021 and 2022 for a total of 24 sessions. Admission was rolling. The sessions were in-person, lasting 45 min. The sessions were offered by one of Mandela Yoga’s Spanish-speaking trained MYP peer facilitator (referred to here as PF). PF was formerly incarcerated and is in recovery, and is now a MYP teacher trainer. PF would often arrive 30–60 min early to the class to lead a men’s circle or help out the participants in other ways and stayed after class to be available to check-in, which he reported was an important element for integration and healing.

Most of the men were from Borinquen (now known as Puerto Rico) and/or identified as Black and/or Latino. This was a population at high risk for overdose; PF estimated that most or almost all of the regular participants had reported heroin or other opioid use in the past. PF reports that at least 15 participants in total attended at least one Mandela Yoga class. PF reports that participants represented the full adult age range, including some young adults (18–25 years old), the general adult age range (26–64 years old), and older adults (above 65 years old) The reentry services classes were attended on average by 1–6 participants each week. Completers were invited to attend a paid Mandela Yoga teacher training. For eligibility for the teacher training, completers were defined as those who attended at least 8 of 12 or 66% of the sessions since their start date. At the 8-month mark, when certificates and a small cash award were offered for 100% attendance, 6 individuals (40%) had attended at least 8 of 12 sessions offered since the start of the program. Of those, 2 (13%) individuals attended all 12 sessions. The 6 individuals who attended at least 66% of sessions were invited to the paid Mandela Yoga teacher training. Of the 6 who began the paid teacher training, 4 (66%) successfully completed it. The reentry services unit coordinator shared via email at the 8-month mark:

*The reentry program had a positive experience with Mandela Yoga. Participants who fully engaged in the 12-[session] program benefited both health-wise and financially. The program provided employment opportunities for those who successfully completed the 12 weeks and were interested in becoming peer Mandela Yoga facilitators. Our relationship with [PF] was excellent; he was able to connect with the reentry participants and also learn from them.*

The FQHC supported access to SUD treatment, primary medical care, and behavioral health care. Of those who receive care at the FQHC, 80% reside in the city where the FQHC is located, which has approximately 90,000 residents, 50% identifying as Hispanic only and 15% identifying as Hispanic plus another ethnicity. Opioid overdose prevalence rates in 2019 for this community were the highest in the state of Massachusetts and were 9 times higher (46.5 opioid related deaths per 100,000 people) than the closest White-majority community it borders (5.7 per 100,000) (61). Of those seeking substance use treatment who provided a toxicology test at the clinic, 66% had evidence of opioid use, including 62% with heroin/fentanyl use (61). In addition, in 2021, 83% of all toxicology tests collected from the community that year demonstrated the presence of fentanyl (62). Of the 68,000 patients that the FQHC serves, 80% are POC, and 70% are best served in a language other than English. To date, 80 participants have been served in some capacity by the reentry services since 2022.

### 3.2 Reflections from PF and PFA

In their respective interviews, PF and PFA reflected on their background and early life experiences growing up in Borinquen (now known as Puerto Rico). They touched upon on a wide range of topics, including their initial connection to martial arts (PFA) and yoga (PF), ancestral stories, experience with gangs, living on the street, experience with drugs, life in prison, and the mind–body and community trauma and stress stemming from these aspects of life. They spoke to their strengths, supports, healing, inner and family wisdom.

PF and PFA also discussed how they came to MYP and their progression within the program. Namely, PF came to MYP after participation in a men of color yoga teacher training in 2020, during which he met the MYP founder, Mr. Thomas, who was a guest speaker. PF took the MYP teacher training because he wanted to help his community access the kind of healing he had through yoga, spiritual and psychological inner work, and men’s groups. Since then, he has taught hundreds of MYP sessions across a variety of community implementations for all ages. PFA attended the entire MYP program at the FQHC reentry services. He had been incarcerated for 30 years. After the reentry services program, PFA participated in a 30-h Spanish-language Mandela Yoga teacher training, after which he became a peer facilitator apprentice with PF.

### 3.3 Key themes from the interviews

PF and PFA repeatedly touched on four key themes that evoke Mandela Yoga’s promise for addiction and trauma recovery for POC:

(1) Breath and Mind–Body Connection Leads to Presence
(2) Consistency
(3) Peer Connection (around shared experience of oppression and peer transmission of presence, hope, and love)
(4) Agency and Positive Action

These themes are expanded upon below and illustrated through excerpts shown in Tables 3, 4.

**Table 3:** Theme 1 (Breath/Mind–Body Connection Leads to Presence) and Theme 2 (Consistency).

Theme	Excerpt	Speaker-Quote#
1. Breath/ Mind–Body Connection Leads to Presence and Consistency	*That’s something you will for sure learn…it will stay in your body after you do the 12 weeks…breathe with [a] purpose…I feel like, [the mind and body are] kinda all connected. Like when you are anxious or something you are going to think either to give power to the thought or you going to think about that tool, it’s going to kick in. When it comes consistently, instead of you giving power to those thoughts…the breath is going to show up. Now that becomes your first option. To breathe. So once you become one with the breath, it’s not just your brain thinking about the breath, you are actually doing it.* …*Even if the first time it takes me 100 breaths to regulate my system at least that thought came first. Now I’m present in my thoughts in my body, And I notice and see what’s happening and I can do something about it.*	PF-1
1. Breath/ Mind–Body Connection Leads to Presence	*PFA: Respirando es vida y life es breathing, y previene lastimarnos*. Interpreter: Breathing is life and life is breathing, and prevents us from getting hurt. *PFA: You know sometimes depression is coming, but if you think like breathing, everything in peace coming back to you.*	PFA-1
1. Breath/ Mind–Body Connection Leads to Presence	*The breath and consistency brings you back to reality. You can realize what’s happening in your brain, and you can do something about it. And you realize what you feel in your classes feels good.*	PF-2
2. Consistency	*It was the consistency that I had with Mandela…every week. Sometimes 2, 3 times a week. I was present, for a year…I’m not saying that’s what you need, because from the beginning it was already changing me…I feel safe….I was able to actually see clearly where I was, stay present and consistent.*	PF-3
2. Consistency	*PFA: Yeah. My sponsor me ayudó en todo.* Interpreter: His sponsor helped him with everything. PFA: *I got here with Mandela Yoga for a year and a half…Pero yo practicó con (PF), every week. No matter about it, every week, every week…”*	PFA-2

**Table 4:** Theme 3 (Peer Connection) and Theme 4 (Agency, Empowered Choice, and Action).

Theme	Excerpt	Speaker-Quote#
1. Breath/ Mind–Body Connection Leads to Presence 3 Peer Connection	*PFA: …an old man, Mexicano…He had a problem with one leg, y me decia a mi en espanol, que le dolia.* Interpreter: …he would tell him in Spanish that it was hurting, the leg was hurting. *PFA: Y yo le decia a el que tranquila, respira profundamente…y pon tu cuerpo en forma derecha. Your shoulders en orden…Y ponte a pensar en algo bonito* Interpreter: I told him, take it easy, breathe deeply, put your body in a straight line and think of something nice. *PFA: Y poquito a poquito vas moviendo la pierna que esta afectada…Muevala, subela… not too much, hacelo con calma…Siempre recordando de que respirando es vida* Interpreter: And little by little start moving the leg that is bothering you. He says if you do it slowly and calmly, it will work, your leg will work….And always reminding them that breathing is life. *PFA: [inaudible]* Interpreter: … And you learn that you can use the mind without using the leg… he says, once you conquer it in your mind, you start doing slowly and reacting with your body. *PFA: Y va sentiendo este paz y amor, dentro de tu organismo, dentro de tu shoulder, muscles; y sentiendo este paz hice que haces algo bueno.* Interpreter: And you feel peace and love in your body, in your organs; and this peace makes you feel the desire to do something good… and you move the leg, and you can see results. *PFA: Y me dicen “next week next week”…Y “next week,” cuando he see me, he hug me; y digo “que pasa?” Y me dice “me siento de nuevo”* Interpreter: Interpreter: They tell him, “next week you talk to me” He said that the following week he hugged him and he said thank you and he said wow, I feel like a new man. *PFA: Y yo digo a el “siempre piensa con calma lo que tu puedes hacer”; Lo piensas con calma, y lo vas haciendo con calma…y todo lo que piensas, lo puedes hacer.* Interpreter: He tells them to think about it first and conquer it in your mind, and then your body will follow. *PFA: He no longer used a cane.*	PFA-3
1. Breath/Mind–Body Connection Leading to Presence 3 Peer Connection	*When you feel that No [from the embodied consent practice] in your body, you can use it. When we slowly come up [from a forward fold], we can give our organs a massage. I give them a better understanding of being present, noticing the body and how it feels, we are giving the body what it needs. We’re starting with giving a hug that day that we have not received. We are the medicine. We really have to give ourselves what we need. This is the tool that we are giving you in Mandela.* *Just like we are scooping from the earth what we need, and putting back what we do not need to carry.*	PF-4
3. Peer Connection	*[They] saw that I was always trying to help. I brought friends to help them with housing issues and things like that.…the little position that I have and the little bit that I could. I used to go 45 min earlier to run a men’s circle…Or…bring my clippers and give them free haircuts, because I knew they could not afford it. When I enter the room…I see my people, people of color… Sometimes I could see [in them], old relatives, my mom, or family members…you see people you know not stressing to get ready for the yoga class. But smiling and going to say “Hi” to another human being. Actually acknowledging the person. That’s something I do not feel when I go to regular classes. The beauty about Mandela is that it’s made for all body types. You have people standing and sitting; you can do it either way. You do what you can or simply you just visualize. They are not going to feel rejected…there’s a position for everybody.*	PF-5
3. Peer Connection	*PFA: Yo me siento como satisfecho y siento amor para la gente más mayores que yo.* Interpreter: He says he feels satisfied, and like a love for these people that are older than him. *PFA: Porque ellos dan testimonio que la forma de cómo hacemos la yoga y de que hablamos, que es una forma de amor así a ellos* Interpreter: They are a testimony of the way they do the yoga and the way they talk about this, he says it’s like a form of love and respect toward them. *PFA: …y queremos que siempre piensen que hay una persona entre ellos que pueden amar y confiar. Y mandela yoga project ha hecho eso, y yo, mucha gente me llaman a mi, messages… en mi telefono…* Interpreter: He wants people to know that this one person they can call if they need. And this program has done that for him– and he gets a lot of calls.	PFA-4
3. Peer Connection	*…I was just lucky enough to be granted an opportunity in life…and that I have the opportunity to help my people….As many tools as I’m teaching them, I know I’m going to learn something from them. I always go to every single person and make them feel seen, shake their hands.*	PF-6
3. Peer Connection	*We are all related, from the 5–10% who survived, just like all the roots in the earth, we just connected like that. Even if we might not know our history, or if we do not remember the why. But our bodies remember, our DNA knows. It’s almost like an urgency of giving the hand that we were hoping to receive once that never came.*	PF-7
3. Peer Connection	*What is consistently present in the peer facilitator, in the community, that was voiced by the peer facilitator, that became the participant’s reality.*	PF-8
4. Agency, Empowered Choice and Action	*[Mandela Yoga] helped me to keep myself strong so I would not make those same old choices. I had new tools. I did not go off to get drunk or go off to find a girl, none of the same habits, the same patterns that I did my entire life. Before I used to think I did not have options…I see my life with Mandela with No and Yes and Maybes* ^1^ *. I get to breathe more. I get to sit back and I understand. And I know now that I always have an option. I can always give myself that option even if I do not get it from the outside.*	PF-9
4. Agency, Empowered Choice and Action	*PFA: [They wanted to take me to the hospital, but I said] No no no, leave me alone… si, yo sabía que como yo entre, yo saldría* Interpreter: …The way he went in, he wanted to get out [of the addiction] *PFA: Y me decian “papi toma, toma, toma. No queremos verte así”* Interpreter: People would tell him “Papi, drink, drink, take this, we do not wanna see you like this” *PFA: Yeah. Mi mamá, mis hermanos, gente en la calle…[were praying for him]* Interpreter: He says, his mom, siblings, people in the streets were praying for him. *PFA: …todo en la vida tiene un principio y un final* Interpreter: … Everything in life has a beginning and an end. *PFA: Si tu quieres terminarlo, lo terminas. Y si tu no quieres, pues te mueras.* Interpreter: If you wanna finish it, you finish it. And if you do not want to, well then you die.	PFA-5
4. Agency, Empowered Choice and Action	*PFA: Y yo, todos estos años que he estado preso y ahora tengo dos años fuera, me siento dichoso, como renovado, una vida sana, limpia y pura [inaudible].* Interpreter: He’s been out for two years after being in jail for that long, and now he feels like he is blessed and he has this way of living that is healthy, clean and pure. You know, he wants to do good, he wants to be good. *PFA: Mandela Yoga me da la oportunidad de yo hablar y me hace sentir seguro de que con Mandela Yoga puedo ir a muchos sitios y puedo ayudar a mucha gente en muchas formas.* Interpreter: Yes, Mandela Yoga is giving him the way to speak, the opportunity to speak. And it makes him feel secure that with Mandela Yoga he can go to many places and help other people in many ways. *PFA: Especialmente hoy en dia, los jovenes necesitan alguien que les hable de que son los resultados de vivir en esta manera en el futuro. Ser un apoyo* Interpreter: Especially today, the youth, they need someone that can talk to them, and tell them what are the results of living that type of life. To give them support. *PFA: Como yo digo, yo estoy aquí. Yo con Mandela digo “I am here…” Todo es un dia a la vez.* Interpreter: Like he says, I am here. With Mandela I say “I am here.” Everything is one day at a time.	PFA-6
4. Agency, Empowered Choice and Action	*[In the teacher training], we talk about our roots and we touch on …our family…the tools that we lack. …I think this is my 4th time, the training. And every time I get rid of something. …even when I participate as a teacher I feel like a student every single time….and that just, like 7 or 8 years of actually taking care of myself. … So what about these people, they never had the chance to do it. If it took me this long and I had the time to grieve and I had the time and the money to sit back and grieve. Is something other people do not have….This is why I started this. The reason I started my own personal healing and I wanted to do the work is because of Mandela Yoga.*	PF-10

PF is referring to the Embodied Consent practice, which supports exploration of how “No,” “Yes,” and “Maybe” feel and can be expressed through the body.

#### 3.3.1 Theme 1: Breath and Mind–Body Connection Leads to Presence

PF spoke early in the conversation about a profound transformation in his relationship to breathing and using the breath to become present and to regulate his nervous system. He speaks about using the breath to make a different choice in the face of emotional challenge (Table 3, Quote PF-1). He speaks not just of a passing awareness of breathing, but of “breathing with a purpose,” “becoming one with the breath” and the path to presence that makes that possible. PFA highlights an essential reciprocal relationship – “breathing is life and life is breathing” – and a protective quality of breathing; “prevents us from getting hurt” (Table 3, PFA-1). He also talks about emotional challenge – depression – and through breathing, peace *coming* back to you (PFA-1).

#### 3.3.2 Theme 2: Consistency

PF talks about consistency (PF-2) “the breath and consistency brings you back to reality,” (PF-2). He speaks about his first year of practicing Mandela Yoga, “…every week. Sometimes 2, 3 times a week. I was present, for a year,” (PF-3) and this consistency allowing for higher-level cognitive awareness (“realize what’s happening in your brain”) as well as a possibility of acting with choice (“and you can do something about it”) (PF-2). PFA on practicing consistently with PF (PFA-2): “Pero yo practicó con (PF), every week. No matter about it, every week, every week….”

#### 3.3.3 Theme 3: Peer Connection (around shared experience of oppression and peer transmission of presence, hope and love)

In describing the consistency with the breath and mind/body connection leading to presence, they both spoke of the essential and catalyzing impact of the peer’s role: to recognize and communicate the shared experience of oppression, and Mandela Yoga being a container for this process, making possible an eventual change in the way this burden feels in the mind and body (Table 4, PFA-3, PF-4). PF described his current working model for the role of the peer: The peer facilitator speaks to the power of the mind–body connection and cues awareness of the breathing as well as the postures whose specific purpose is to heal the mind–body from the trauma of racism and oppression, while fostering a sense of belonging to community and to the earth (PFA-3, PF-4). Through the consistency in relational connection, the peer facilitator also transmits his understanding for the struggle that the participants endure(d), and his own path of healing, recovery, and peace (PF-5, PF-8). Slowly participants start to look to the breath and body (which is usually a contrast from the previous numb, shameful, confusing or adversarial relationship with the body), supported by the peer’s consistent voicing of it and the community support for adopting a new reality in oneself. This is a reality that was once lost and taken from them, and has been forgotten (PF-7). Through the integration of breath, body, the example of the peer facilitator, and consistent supportive presence, they start to develop an experiential conviction that they can adopt a new reality toward peace (PFA-3).

They speak of a mutuality in which they themselves also heal and learn and grow from this process (PFA-4, PF-6). PFA and PF both spoke of the qualities of love, family, and kinship they feel with their participants, which fuels their calling to serve (PF-5, PF-6, PF-7, PFA-4). PF: “It’s almost like an urgency of giving the hand that we were hoping to receive once that never came.” PFA spoke in numerous ways about being a community servant. The following are some of the examples which capture the importance of the transmission of presence, hope, and love for PFA. His mother was a major inspiration for him. She worked as a chef in a school cafeteria in Borinquen (Puerto Rico) for 33 years; she would make sure everyone was cared for and fed; this moved PFA immensely. In jail, PFA found many ways over the 30 years to serve his fellow peers. He would share leftovers and arrange for special meals for his peers and the staff; he spoke of them as his children. PFA’s relationship to God is another major source of inspiration and deep love for him; he spoke extensively on this topic, and while it is beyond the scope of this manuscript, it can be summarized through the way he describes his deepest purpose: “Love, love, love.” He connected the opportunity to serve through Mandela Yoga as a way to continue the stream of service that imbued his positive actions from the past. He gave the example of his recent apprenticeship work in Mandela Yoga where he took the initiative to informally interview participants in his community about their experience with the program in order to build connections, witness them and uplift their voices.

#### 3.3.4 Theme 4: Agency, Empowered Choice and Action

PFA and PF speak of the synergistic relationship between the first three themes leading to a sense of increased agency in difficult situations, and more consistent awareness of the possibility to make empowered choices to take positive actions. PFA’s relationship with his mother, his relationship with Jesus, and his relationship with himself figure prominently in his sources of agency prior to arriving at Mandela Yoga; all are present during his choice to endure his symptoms of withdrawal from heroin, cocaine, and alcohol when he first arrived to jail (PFA-5). PFA weaves these sources of agency into the present (PFA-6) in describing his calling to serve his community “I am here.” PF speaks of choices, how choices feel in the body, and how he could integrate those choices into deeper layers of life (PF-9). He speaks of the iterative process of deep emotional and spiritual healing, and he voices this when reflecting on the future of Mandela Yoga and the potential for systemic change (PF-10):

*So what about these people, they never had the chance to do it. If it took me this long and I had the time to grieve and I had the time and the money to sit back and grieve. Is something other people don't have ….This is why I started this. The reason I started my own personal healing and I wanted to do the work is because of Mandela Yoga.*

## 4 Discussion

Mandela Yoga was implemented in a health center-based community re-entry program with the aims of prevention of overdose and reducing recidivism. This project demonstrated feasibility in a primarily Spanish-speaking community with men of color. Future research is needed to continue to optimize the program and understand how to further improve engagement and retention in this population that has high levels of attrition. The qualitative evaluation provides depth to the understanding of the ways MY supported recovery and healing in this setting, and may support addiction and trauma recovery for future implementations.

Given that typical retention rates for this post-incarceration reentry population are 30–40% (63), consistent attendance over 12 weeks for 6 of 15 participants (40%) with high acceptability (6 attended at least 66% of sessions and 2 attended 100% of sessions) is on par with other successful post-incarceration interventions, and the inclusion of a mind–body component did not detract from engagement compared with other post-incarceration group interventions. A novel addition in this program is that the 6 participants who attended at least 8 of 12 sessions were invited to a paid teacher training following the 12-week intervention, and 4 of the 6 completed the teacher training. One opportunity for increasing engagement in future implementations could be introducing paid teacher training opportunity and initiating it at an earlier timepoint during the 12-week program. This potentially could incentivize participants to receive payment for attendance, reduce the impact of any financial barriers to attendance (e.g., transportation costs), and could open a pathway to employment earlier in the program. Considerations for future implementations could also include how to work with the health center to recruit a higher volume of participants. In subsequent implementations, MYP experienced success through offering Mandela Yoga at community events, partnering long-term with residents of the city where the intervention was being offered, as well as with community-based leaders who could serve as champions for the population being served. Considerations for sustainably going to scale include support for the nonprofit to achieve a steady stream of funds through government, health system, and philanthropic sources so that programmatic partners and peer facilitators may be able to achieve stability in their MYP programming and employment, respectively.

The implementation at the FQHC re-entry services was focused on adult men who were at high risk for overdose from opioids. One limitation of the study is that we did not have information on the number of young adults (18–25 years old) attending the program. Since young adults have historically low attendance and retention in substance use treatment compared with older adults, more research is needed into the feasibility and impact of MY for young adults at risk of substance use and incarceration (64). Readiness for change, a different conception of treatment and recovery, and external factors may play a role in the difference between younger and older adults’ adoption of substance use treatment (65, 66). Yoga may support substance use outcomes for at-risk youth through emotion regulation including improving distress tolerance and reducing stress (67). Further research is needed to understand how peer-led yoga programs could influence substance use and recidivism among at-risk young adults, and to identify effective implementation models for delivering these interventions in real-world settings.

Additional accounts of Mandela Yoga by others who have been substantially impacted by substance use in the post-incarceration setting would strengthen our understanding of which aspects may be specifically supportive for re-entry and opioid use disorder, and which aspects may be less supportive. The four areas which PF and PFA identified as being core components of their Mandela Yoga experience (Breath and Mind–Body Connection Leads to Presence, Consistency, Peer Connection, Agency and Positive Action), point to potential mechanisms for the impact of Mandela Yoga on recovery and reducing recidivism. Both PF and PFA framed their recovery process in Mandela Yoga in terms of healing the impact of racial and gang/violence-based trauma on their nervous systems, which for PF, supported recovery from his addictions. They both spoke of the role of trauma and poverty on their perceived lack of choice and agency in their initial and prior involvement with substances. There is an established association between drug use and discrimination among Latino men (5), especially those who were US-born. Other Indigenous communities have connected the role of poverty, trauma, and race and discrimination with alcohol use disorder (4). PF and PFA were also each able to find their own voice as peer facilitators; PF speaks to his path with men’s circles, grieving, greeting each person, giving him the opportunity to lend a hand to others that he did not have, playing Taino flute; PFA speaks to embodying and serving through his inspiration from his mother, from God, from his calling to be a community servant. Building community is spoken of as an alive, dynamic, and responsive process (e.g., PF brought in his barber’s clippers, and brought in community members in to help with housing; PFA speaks of sharing food in jail, interactions with the Mexican older man, knowing how to talk to many different types of people).

We propose that MY, like other mindfulness-based and movement-based embodied contemplative trainings (26) supports interoceptive awareness and nonjudgment, curiosity and sustained attention to present moment sensations, emotions, and urges, and self-regulation. Through these pathways, MY may support emotion regulation through, for example, reduced rumination (68), in the context of chronic stress and trauma history. In addition, MY may also support psychological adjustment in the context of trait neuroticism and/or associated emotional challenges such as alexithymia (69); given the importance of these psychological features in the general population, and among people experiencing mental health disorders in the context of trauma and post-incarceration (70, 71), it is important to look further into the psychological mechanisms by which MY may reduce emotional distress. In addition, given the potential role of disrupted attachment—stemming from adverse childhood events or maltreatment, which are common in the incarcerated population and impact personality development and coping behaviors (72–74)—the ecological approach featured in MY may facilitate attachment healing. By considering participants’ identities, culture, and experiences with oppression, and by integrating community-based support through the peer facilitator, this approach may foster a more positive relationship with oneself, the peer facilitator, and the group (75, 76). More research is needed into the mechanisms of MY that may contribute to reducing cognitive and emotionally distressing patterns associated with mental health disorders, and increasing positive attachment.

The participatory methods used to describe this community implementation and the emerging key themes may be instructive for design and evaluation of other community-based recovery programs (59) to improve cultural relevance, recruitment and retention strategies, and community partnership. The community perspectives in the present study helped highlight the importance of the mind–body connection and peer connection as a support to recovery, rather than content or themes explicitly related to substance use, craving, avoidance, or addiction. The peers chose to reflect on how Mandela Yoga increased access to fulfilling (e.g., peace in the body and mind, serving others, emotional healing) and protective (e.g., breath and community) capacities and experiences in the face of challenge, rather than removing or decreasing difficult or depleting experiences.

These insights may point to the potential for Mandela Yoga to increase recovery capital—the volume of internal and external assets supportive to an individual’s recovery (77), an element that has been identified as important in the recovery journey in general, and with specific elements supporting recovery in the post-incarceration setting. Qualitative evaluation of post-incarceration interventions has provided valuable information about the importance of peer support to increase recovery capital. Interventions that feature peer connection and social support have demonstrated efficacy and acceptability in the post-incarceration setting (63, 78, 79), particularly in the context of substance use. Mindfulness, practice, and peer support were facilitative of recovery capital in another recovery setting (80). Particularly for racial/ethnic minorities (79, 81), cultural and community recovery capital may be particularly important domains (82). Yet, there is an overall dearth of studies on successful treatment programs focused on racial/ethnic disparities in post-incarceration settings. Peer-led Mandela Yoga may be well-suited to be more culturally resonant for racial/ethnic minorities than existing models of care, particularly as integrated into group-based recovery treatment, such as in office-based opioid treatment with buprenorphine (30, 83, 84). Integrating a culturally responsive mind–body component paired with culturally-relevant stigma reduction education about the benefits of medication for OUD in parallel with opportunities for buprenorphine medication-assisted opioid group-based treatment may decrease medication-associated stigma (85) and increase engagement by reducing withdrawal and craving symptoms (85). Further research is needed to understand the specific contributions of Mandela Yoga to recovery capital and as a recidivism treatment for communities of color in the post-incarceration setting, and as an adjunct to medications to increase recovery capital during treatment with medications for opioid use disorder (84).

The current community case study should be considered in the context of a few limitations. First, the case study did not report on the characteristics or experiences of the other participants in the implementation. This information was not accessible to the authors. In addition, the authors did not have access to data on the impact of Mandela Yoga on opioid overdose prevention, which was the focus of the overarching reentry service program. While 15 participants attended one session of Mandela Yoga, it was well-adopted by 6 participants; more exploration is needed to understand the barriers to engagement for the non-completers. Future research is needed to continue to optimize the program and understand how to further improve engagement and retention in this population that typically has high levels of attrition. Finally, the current report employed a thematic analysis within an iterative CBPR framework, which emphasized power-sharing (59). Another approach for future research could include interpretive phenomenological analysis, which could capture the lived experiences more closely and more vividly (86).

## 5 Conclusion

At the heart of PF and PFA’s experiences of Mandela Yoga sat a familial calling to help restore in oneself and for one’s community, through breathing and presence in the mind and body, what was once lost, oppressed, taken, and forgotten. The Mandela Yoga intervention offered PF and PFA and their peers the opportunities to reclaim a peaceful, empowered relationship with the breath, body, community, and earth in the face of oppressive conditions. Consistency of purposeful breathing and strengthening the mind–body connection combined with peer transmission of presence, hope and love allowed them to pave a path of agency to access empowered choice and action in the face of emotional challenge, craving, and despair. For mind–body interventions to be able to support deep, lasting change for those with oppressed identities, they may need to expand the scope to acknowledge and help address the whole ecology in which the individual is a part (36, 87). Thus far, most MBI research and interventions focus primarily on the internal, individual experience. We propose that Mandela Yoga, as facilitated and related by PF, is a mind–body-community intervention that builds on and goes beyond what is offered by existing MBIs. It is a multifaceted intervention that is peer-led, trauma-informed, community-oriented, and culturally-responsive. We posit that recovery programs that can work directly with the seeds of addiction and trauma, and nourish the seeds of witnessing and connection, cultural strengths, and peer examples of recovery and service through embodied practice, could have great promise for communities of color in recovery. As PF says, “But our bodies remember, our DNA knows. It’s…an urgency of giving the hand that we were hoping to receive once that never came.”

## Data Availability

The raw data supporting the conclusions of this article will be made available by the authors, without undue reservation.
