# Supplementary material for: Mandela Yoga: a community case study for a post-incarceration reentry service for men of color in recovery
Source: Front Public Health. 2025 May 9;13:1514946. doi: 10.3389/fpubh.2025.1514946 (PMC12098574; doi:10.3389/fpubh.2025.1514946)
Supplement: Supplementary file 1 [file Table_1.docx]

Supplementary Table 1. Key Dimensions of Mind-Body Interventions Shared by Mandela Yoga

|  | **Elements of Mindfulness/Yoga Programs** | **Mandela Yoga Associated Thread** |
| --- | --- | --- |
| **Essential Properties of Yoga Questionnaire**(1) | Acceptance/compassion | Thread 8 |
|  | Breathwork | Thread 5 |
|  | Physicality | Threads 5 and 7 |
|  | Postures, active | Threads 5 and 7 |
|  | Postures, restorative | Threads 5 and 7 |
|  | Body locks | *(Not present)* |
|  | Body awareness | Thread 6 |
|  | Mental & Emotional awareness/release | Thread 1 and 6 |
|  | Health benefits | Thread 5 |
|  | Individual attention | Thread 1 and 2 |
|  | Social Aspects | Thread 2 |
|  | Spirituality | Thread 4 |
|  | Meditation & Mindfulness | Thread 6 |
|  | Yoga Philosophy | Threads 5-8 |
| **Movement-Based Embodied Contemplative Practices**(2) | Movement (Intentional induction or disinhibition of overt or subtle) | Threads 5-7 |
|  | Embodied explicit emphasis on attending to interoceptive, proprioceptive and kinesthetic qualities of experience. They also use concepts such as “being in one's body” to encourage an embodied experience of the self. | Threads 6-8 |
|  | Contemplative (Self-awareness, disciplined process of becoming reflectively attentive to experience, suspending habitual thoughts/redirecting experience back to body sensations) | Threads 3-8 |
|  | Dyadic Contemplation | Thread 2 |
| **Core Components of Mindfulness-Based Programs**(3) | 1. Is informed by theories and practices that draw from a confluence of contemplative traditions, science, and the major disciplines of medicine, psychology and education | Threads 1-8 |
|  | 2. Is underpinned by a model of human experience which addresses the causes of human distress and the pathways to relieving it | Threads 1-8 |
|  | 3. Develops a new relationship with experience characterized by present moment focus, decentering and an approach orientation | Threads 5-8 |
|  | 4. Supports the development of greater attentional, emotional and behavioral self-regulation, as well as positive qualities  such as compassion, wisdom, equanimity. | Threads 5-8 |
|  | 5. Engages the participant in a sustained intensive training in mindfulness meditation practice, in an experiential inquiry-based learning process and in exercises to develop insight and understanding | Threads 5-8 |
| **Common Elements for Coping and Empowering in the context of Racial Stress and Trauma**(4) | Self-Care | Threads 1-8 |
|  | Self-Compassion | Threads 1-8 |
|  | Social Support | Thread 2 |
|  | Mindfulness | Threads 6-8 |
|  | Psychoeducation and Cognitive Restructuring | Threads 1-8 |
|  | Cognitive Defusion | Threads 1-8 |
|  | Identity-Affirming Practices and Development of Racial/Ethnic Identity | Threads 1-4 |
|  | Expressive Writing | *(Not present)* |
|  | Social Action and Activism | Threads 1-4 |
|  | Psychedelics | *(Not present)* |

(1) Park CL, Elwy AR, Maiya M, Sarkin AJ, Riley KE, Eisen S V, et al. The Essential Properties of Yoga Questionnaire (EPYQ): Psychometric Properties. Int J Yoga Therap. 2018 Nov;28(1):23-38;
(2) Schmalzl L, Crane-Godreau MA, Payne P. Movement-based embodied contemplative practices: definitions and paradigms. Front Hum Neurosci [Internet]. 2014;8:205;
(3) Crane RS, Brewer J, Feldman C, Kabat-Zinn J, Santorelli S, Williams JMG, et al. What defines mindfulness-based programs? The warp and the weft. Psychol Med. 2017 Apr 47(6):990-999;
(4) Holmes SC, Zare M, Haeny AM, Williams MT. Racial Stress, Racial Trauma, and Evidence-Based Strategies for Coping and Empowerment. Annu Rev Clin Psychol. 2024 Jul 12;20(1):77–95.
